# Supplementary material for: The role of a structured community health worker network in achieving malaria elimination goals in the Dominican Republic: An impact evaluation amid COVID-19 disruptions
Source: PLOS Glob Public Health. 2024 Dec 18;4(12):e0003648. doi: 10.1371/journal.pgph.0003648 (PMC11654942; doi:10.1371/journal.pgph.0003648)
Supplement: Attachment — Submitted filename: Response to Reviewers (1).docx [file pgph.0003648.s001.docx]

**Nicole Michelén Ströfer**

**Clinton Health Access Initiative**

**michelenstrofernicole@gmail.com**

**10/23/2024**

**PLOS Global Public Health**

Dear Reviewer,

Thank you for the insightful comments and the opportunity to improve our manuscript. We have carefully considered each suggestion and have revised the manuscript accordingly. Below, we provide a response to each comments raised. Additionally, we implemented one minor revision in the authorship order to better reflect author’s contributions.

**Reviewer 1**

**Comment #1: Characteristics of COVID Lockdown**

***Reviewer’s comment:***
"It would be helpful to have more detail on government guidelines during the COVID lockdown. If a household visit does not take place, possible reasons are 1) government guidelines prohibiting household visits, 2) government allows household visits, but CHWs prefer not to make them, 3) CHWs make household visits, but householders do not allow CHWs to visit/enter the house. If there were government prohibitions on household visits, on what date did they come into effect, and when did they end?"

***Response:***
Thank you for your insightful comment regarding the characteristics of the COVID lockdown and its impact on household visits. We agree that it is essential to clearly distinguish between government restrictions and individual or CHW preferences during this period. To address this, we have made edits to clarify the nature of the suspension of CHW household visits during the lockdown and explained additional factors that influenced household visits once CHWs were allowed to resume their work.

In the revised manuscript, we clarify that during the COVID-19 lockdown (March-September 2020), government guidelines prohibited household visits entirely, pausing all CHW activities. After the lockdown, as restrictions eased (September 2020-February 2021), CHWs were allowed to resume limited household visits (2-3 days per week) based on the availability of personal protective equipment (PPE). During this period, the number of household visits was further affected by the willingness of community members to allow CHWs into their homes, as well as the CHWs’ own comfort levels in performing visits during the pandemic. Finally, administrative delays in contract renewals led to further suspensions of CHW work from February to July 2021, when contracts were renewed.

**Comment #2: Characteristics of the CHW Program**

***Reviewer’s comment:***
"The paper tells us almost nothing about the CHWs, or the larger CHW program. What other responsibilities do CHWs have? Are they malaria-specific, or do they have a range of responsibilities and assigned tasks? Do they report to the malaria program, or to a national CHW program? How are they recruited? What training do they receive (e.g., 2 weeks of basic training in classroom followed by 4 weeks of close supervision)?"

***Response:***
Thank you for your feedback. We agree with your assessment and have added a more comprehensive description of the CHW program, including their recruitment process, training, contractual obligations, and broader responsibilities.

We have clarified that the CHW network is malaria-specific, designed to strengthen malaria detection and treatment through proactive household visits. CHWs are recruited based on community recognition, literacy, place of residency, and other specific criteria. They undergo five days of classroom-based training that covers both theoretical and practical components, after which they are selected based on their training performance. During their first week of service, CHWs are closely supervised in the field by Ministry of Health personnel. CHWs are responsible for visiting 100-200 households per week, testing symptomatic individuals using rapid diagnostic tests and blood smears, and providing directly observed therapy for three days to those who test positive.

**Other minor changes:**
We would also like to inform you that we have made a change in the authorship order by placing Justin M. Cohen as last author instead of Harriet G. Napier, since he is the senior author. This modification has been agreed upon by authors. We believe this new order better reflects their respective contributions to the manuscript.

We hope the revisions and additional details address your concerns. Thank you once again for your valuable feedback. We look forward to your response and hope that the revised manuscript now meets the journal's requirements for publication.

Sincerely,
Nicole Michelén Ströfe**r**
Clinton Health Access Initiative
